# The Presence, Persistence and Functional Properties of *Plasmodium vivax* Duffy Binding Protein II Antibodies Are Influenced by HLA Class II Allelic Variants

**DOI:** 10.1371/journal.pntd.0005177

**Published:** 2016-12-13

**Authors:** Flora S. Kano, Flávia A. Souza-Silva, Leticia M. Torres, Barbara A. S. Lima, Taís N. Sousa, Jéssica R. S. Alves, Roberto S. Rocha, Cor J. F. Fontes, Bruno A. M. Sanchez, John H. Adams, Cristiana F. A. Brito, Douglas E. V. Pires, David B. Ascher, Ana Maria Sell, Luzia H. Carvalho

**Affiliations:** 1 Centro de Pesquisas René Rachou/FIOCRUZ Minas, Belo Horizonte, Minas Gerais, Brazil; 2 Centro de Pesquisas Leônidas e Maria Deane/FIOCRUZ, Manaus, Amazonas, Brazil; 3 Faculdade de Medicina, Universidade Federal de Mato Grosso, Cuiabá, Mato Grosso, Brazil; 4 Instituto de Ciências da Saúde, Universidade Federal de Mato Grosso, Campus Sinop, Sinop, Mato Grosso, Brazil; 5 Department of Global Health, College of Public Health, University of South Florida, Tampa, Florida, United States of America; 6 Department of Biochemistry, University of Cambridge, Cambridge, United Kingdom; 7 Department of Biochemistry, University of Melbourne, Victoria, Australia; 8 Programa de Pós-graduação em Biociências e Fisiopatologia, Universidade Estadual de Maringá, Paraná, Brazil; Queensland Institute of Medical Research, AUSTRALIA

## Abstract

**Background:**

The human malaria parasite *Plasmodium vivax* infects red blood cells through a key pathway that requires interaction between Duffy binding protein II (DBPII) and its receptor on reticulocytes, the Duffy antigen/receptor for chemokines (DARC). A high proportion of *P*. *vivax*-exposed individuals fail to develop antibodies that inhibit DBPII-DARC interaction, and genetic factors that modulate this humoral immune response are poorly characterized. Here, we investigate if DBPII responsiveness could be HLA class II-linked.

**Methodology/Principal Findings:**

A community-based open cohort study was carried out in an agricultural settlement of the Brazilian Amazon, in which 336 unrelated volunteers were genotyped for HLA class II (*DRB1*, *DQA1* and *DQB1* loci), and their DBPII immune responses were monitored over time (baseline, 6 and 12 months) by conventional serology (DBPII IgG ELISA-detected) and functional assays (inhibition of DBPII–erythrocyte binding). The results demonstrated an increased susceptibility of the DRB1*13:*01* carriers to develop and sustain an anti-DBPII IgG response, while individuals with the haplotype DRB1*14:02-DQA1*05:03-DQB1*03:*01* were persistent non-responders. HLA class II gene polymorphisms also influenced the functional properties of DBPII antibodies (BIAbs, binding inhibitory antibodies), with three alleles (DRB1*07:*01*, DQA1*02:*01* and DQB1*02:*02*) comprising a single haplotype linked with the presence and persistence of the BIAbs response. Modelling the structural effects of the HLA-DRB1 variants revealed a number of differences in the peptide-binding groove, which is likely to lead to altered antigen binding and presentation profiles, and hence may explain the differences in subject responses.

**Conclusions/Significance:**

The current study confirms the heritability of the DBPII antibody response, with genetic variation in HLA class II genes influencing both the development and persistence of IgG antibody responses. Cellular studies to increase knowledge of the binding affinities of DBPII peptides for class II molecules linked with good or poor antibody responses might lead to the development of strategies for controlling the type of helper T cells activated in response to DBPII.

## Author Summary

Vaccines are a crucial component of the current efforts to eliminate malaria, and much of the vaccine-related research on *P*. *vivax* has been focused on the Duffy binding protein II (DBPII), a ligand for human blood stage infection. A high proportion of individuals who are naturally exposed to *P*. *vivax* fail to develop neutralizing antibodies, but the host genetic factors modulating this immune response are poorly characterized. We investigated whether DBPII responsiveness was dependent on the variability of human leucocyte antigen (HLA) class II cell surface proteins involved in the regulation of immune responses. To obtain a reliable estimate of DBPII antibodies, we carried out a longitudinal study, collecting serum from the same individuals over a period of 12-months. The results confirmed the heritability of the DBPII immune response, with genetic variation in HLA class II genes influencing both the development and persistence of the antibody response. HLA class II genotype also influenced the ability of DBPII antibodies to block the ligand-receptor interaction *in vitro*. Computational approaches identified structural specificity between HLA variants, which we propose as an explanation for differences between a good or poor antibody responder. These results may have implications for vaccine development, and might lead to strategies for controlling the type of immune response activated in response to DBPII.

## Introduction

*Plasmodium vivax* infects human reticulocytes through a major pathway that requires interaction between an apical parasite protein, the Duffy binding protein (DBP), and its cognate receptor on reticulocytes, the Duffy antigen/receptor for chemokines (DARC) [1–3]. Although most individuals lacking DARC on their red blood cells (RBCs) are naturally resistant to *P*. *vivax* [1], some infections occur in DARC-negative individuals living in vivax malaria endemic areas [4–6, 70]. So far, no alternative ligand facilitating the binding of *P*. *vivax* to reticulocytes has been identified, which makes the DBP one of the most promising *P*. *vivax* vaccine targets [8].

The importance of the interaction between DBP (region II, DBPII) and DARC to *P*. *vivax* infection has stimulated a significant number of studies on DBP antibody responses (reviewed in [8]). The available data demonstrate that naturally occurring antibodies to DBP are prevalent amongst individuals living in *P*. *vivax* endemic areas, and that these antibodies can inhibit the DBPII-DARC interaction [7, 9–12]. Even though DBPII-specific binding inhibitory antibodies (DBPII BIAbs) seem to confer a degree of protection against blood stage infection [11], the majority of people naturally exposed to *P*. *vivax* do not develop a DBPII BIAbs response [8]. In the Amazon Basin, for example, this inhibitory activity was detected in only one third of malaria-exposed subjects [8, 13]. Similarly, less than 10% of children from Papua New Guinea (PNG) with immunity to malaria had acquired high levels of DBPII BIAbs [11]. Given the significant differences in epidemiology and parasite genetics between the Amazon Basin and PNG, the fact that the DBPII BIAbs response is relatively low but also remarkably stable over time is particularly intriguing.

The reasons for the low immunogenicity of DBPII are not clear, but may be linked to a complex immune response driven by genetic diversity in both the parasite and human populations. Several studies have demonstrated the existence of variant specificity in the natural immune response against DBPII, which has been attributed to allelic diversity [12, 14]. On the host side, recent evidence suggests that host genetic polymorphisms might also affect humoral immunity against DBP [15, 16], with DARC polymorphisms thought to affect the ability of DBP antibodies to block parasite invasion [16]. In a previous study, we demonstrated that the naturally acquired BIAbs response tended to be more frequent in heterozygous individuals carrying a DARC-silent allele (FY*B^ES^), which suggested that gene-dosage effect occurred [7]. In this context, we were interested in determining if DBPII non-responsiveness could be associated with variation in the major histocompatibility complex.

While malaria infection represents a key selection pressure for the human leukocyte antigen (HLA), and has left clear evolutionary footprints on the alleles observed in different countries [17], the association between HLA gene expression and responsiveness (or non-responsiveness) to defined malaria antigens has produced contradictory results [18–21]. Beyond the extreme genetic diversity of HLA class II, which hinders interpretation of the role of HLA on antibody responses elicited during malaria, most studies rely on antibody prevalence data collected at a single time-point in cross-sectional analysis of a population. Since malaria transmission is intermittent and seasonal in many endemic areas, it is possible that antibody levels fluctuate over time such that individuals could appear to be non-responders on some occasions and responders on others [20]. In the current study, we present data of the first ongoing population-based study of the relationship between HLA class II genes and DBPII immune response. The methodological approach included a community-based open cohort study in an agricultural settlement of the Brazilian Amazon, in which 336 unrelated volunteers were genotyped for HLA class II (*DRB1*, *DQA1* and *DQB1* loci), and their DBP immune responses were monitored over time by conventional serology (DBPII IgG ELISA-detected) and functional assays (DBPII BIAbs).

## Methods

### Study area and population

**Fig 1:**
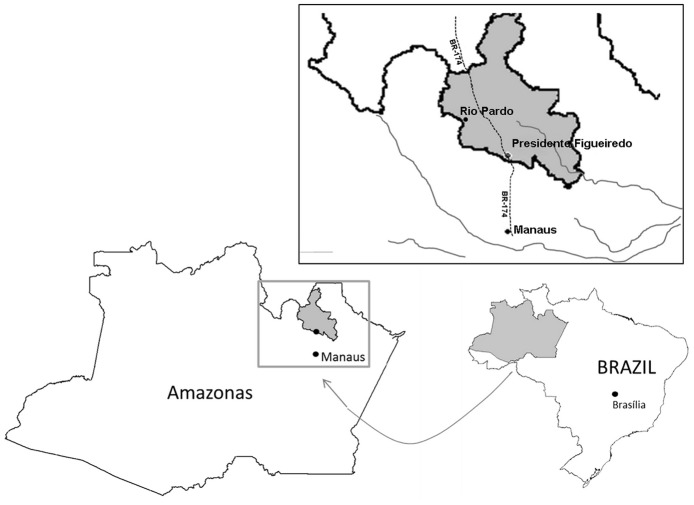
Map of the state of Amazonas, Northwestern Brazil, showing the study site. The Rio Pardo settlement is located within the Presidente Figueiredo municipality (grey area in the inset), roughly 160 km from the state capital, Manaus.

The study was carried out in the agricultural settlement of Rio Pardo (1°46’S—1°54’S, 60°22’W—60°10’W), in the Presidente Figueiredo municipality, located in the Northeast of Amazonas State in the Brazilian Amazon. The Rio Pardo settlement is located approximately 160 km from Manaus, the capital of Amazonas, along the main access to a paved road (BR-174) that connects Amazonas to Roraima (Fig 1). The settlement was officially created in 1996 by the National Institute of Colonization and Agrarian Reform (INCRA) as part of a large-scale colonization project focused on agriculture and wide-ranging human settlement in the Amazon area [22]. The mean annual temperature is 31°C with a humid climate and an average annual rainfall of 2,000 mm per year. The rainy season extends from November to May, and the dry season from June to October. According to a census conducted between September and October 2008, Rio Pardo has 701 inhabitants, most of whom live on subsistence farming and fishing along the tributaries of the Rio Pardo River. The study population was quite stable. Most residents were native to the Amazon region, and their average age of 28 years roughly corresponded to the time of malaria exposure in the Amazon area [7]. In the study area, migration rates were relatively low, as only 28 (8%) of 336 individuals moved out of the village during the follow-up period.

Based on the spleen size of the local children and parasite infection rates, the study area was classified as hypo- to mesoendemic, which is consistent with the general profile of malaria infection for well-established frontier settlements in the Amazon region [23]. The study site and malaria transmission patterns have been described in detail elsewhere [7]. Although *P*. *vivax* and *P*. *falciparum* are transmitted year round, *P*. *vivax* is responsible for about 90% of malaria cases in the region. Similar to other parts of the Brazilian Amazon area [24], a continuous decrease in the number of malaria cases has been reported in the Rio Pardo community; in 2008, the local Annual Parasitological Index (API) was 131 cases per 1000 inhabitants, while in 2009 the API was 54.6 (Health Surveillance Secretariat of the Brazilian Ministry of Health, SVS/MS). In the study area, precarious living conditions, including houses with partial walls and roofs made of tree leaves, increase human-vector contact and reduce indoor residual spraying efficacy [23]. However, while the availability of curative services is limited, a government outpost in the area provides free malaria diagnosis and treatment.

### Study design and cross-sectional surveys

The ethical and methodological aspects of this study were approved by the Ethical Committee of Research on Human Beings from the Centro de Pesquisas René Rachou (Reports No. 007/2006, No. 07/2009, No. 12/2010 and No. 26/2013), according to the Resolution of the Brazilian Council on Health-CNS 466/12. In November of 2008, 541 of the 701 residents of the settlement (77.2%) invited to participate in the study accepted by giving written informed consent, which was also obtained from the next of kin, caregivers, or guardians on the behalf of participating minors.

A population-based open cohort study was initiated in November of 2008, with the following procedures: (i) administration of a structured questionnaire to all volunteers to obtain demographical, epidemiological, and clinical data; (ii) physical examination, including body temperature and spleen/liver size, recorded according to standard clinical protocols; (iii) venous blood collection for individuals aged five years or older (EDTA, 5 mL), or blood spotted on filter paper (finger-prick) for those aged <5 years; and (iv) examination of Giemsa thick blood smears for the presence of malaria parasites via light microscopy. The geographical location of each dwelling was recorded using a hand-held 12-channel global positioning system (GPS) (Garmin 12XL, Olathe, KS, USA) with a positional accuracy of within 15 m. At the time of initial enrollment in the study, 222 out of 541 volunteers had no familial relationships with other volunteers, and were consequently selected for HLA genotype and serological assays.

Six and twelve months after the initial survey, two similar cross-sectional surveys were carried out. In total, 336 unrelated subjects were enrolled in the study, with 222 examined in the baseline cohort, 249 examined during the 2nd survey (June, 2009), and 239 during the 3rd survey (October-November, 2009). A total of 244 (72.6%) subjects had consecutive samples taken, and 156 of these (64%) had samples taken in all cross-sectional surveys (baseline, 6 and 12 month follow-up).

### Laboratory diagnosis of malaria

Malaria infections were diagnosed by microscopy of Giemsa-stained thick blood smears, and by Real-Time PCR amplification of a species-specific segment of the multicopy 18SSU rRNA gene of human malaria parasites. The Giemsa-stained smears were evaluated by experienced microscopists, according to the malaria diagnosis guidelines of the Brazilian Ministry of Health. For Real-Time PCR, genomic DNA was extracted from either whole blood samples collected in EDTA, or from dried blood spots on filter paper using the Puregene blood core kit B (Qiagen, Minneapolis, MN, USA) or the QIAmp DNA mini kit (Qiagen), respectively, according to manufacturers’ instructions. Real-Time PCR was performed as previously described [25].

### HLA genotyping

Molecular amplification of the alleles of *HLA-DRB1*, *HLA-DQB1* and *HLA-DQA1* were performed by the PCR-SSO (polymerase chain reaction, specific sequence of oligonucleotides) technique, with Luminex technology (One Lambda Inc., Canoga Park, CA, USA). Briefly, target DNA was PCR-amplified using group-specific primer sets, after the amplified product was biotinylated, which allowed later detection using R-Phycoerythrin-conjugated Streptavidin (SAPE), and hybridized with microspheres linked to specific conjugated fluorescent probes for each HLA allele group (One Lambda, Canoga Park, CA, USA). The fluorescent intensity varied based on the reaction outcome, with an expected intensity of 1000 or more for positive control probes. Reaction readings were carried out by flow cytometry using Luminex technology (One Lambda). Samples were analyzed through the HLA FUSION software (One Lambda Inc., San Diego, CA, USA).

### Recombinant DBPII and ELISA-detected IgG antibodies

A conventional enzyme-linked immunosorbent assay (ELISA) for total IgG antibodies to DBPII was carried out using a recombinant protein that included amino acids 243–573 (region II) of the Sal-1 DBPII variant, which is highly prevalent in the study area [23]; the recombinant protein was expressed as a 39 kDa 6xHis fusion protein, as previously described [26]. ELISA was carried out as previously described [27], with serum samples at 1:100 and DBPII at a final concentration of 3 μg/ml. The results were expressed as reactivity index (RI), calculated by dividing the reading values of the test (OD values) by the cut-off (mean reading for the unexposed group plus 3 SD, n = 30). Values of RI > 1.0 were considered positive.

### COS7 cells transfections and DBPII inhibitory binding assays

COS7 cells (green monkey kidney epithelium, ATCC, Manassas, VA) were transfected with the plasmid pEGFP-DBPII, which coded for a common DBPII sequence circulating in the Amazon area [13]. Transfections were performed with lipofectamine and PLUS-reagent (Invitrogen Life Technologies, Carlsbad, CA) according to manufacturer’s protocols. Forty-eight hours post-transfection, erythrocyte-binding assays were performed as previously described [10]. Briefly, plasma samples were added at 1:40, and plates were incubated for 1 hr at 37°C in 5% CO2. Human O+ DARC+ erythrocytes in a 10% suspension were added to each well (200 μl/well), and plates were incubated for 2 h at room temperature. After incubation, unbound erythrocytes were removed by washing the wells three times with phosphate buffered saline (PBS). Binding was quantified by counting rosettes observed in 10–20 fields of view (x200). Positive rosettes were defined as adherent erythrocytes covering more than 50% of the COS cell surface. For each assay, pooled plasma samples from Rio Pardo residents characterized as non-responders by ELISA were used as a negative control (100% binding). For this purpose, only plasma that did not inhibit erythrocyte binding was pooled for use as the negative control (usually, 10 plasma samples/pool). The positive control included a pool of plasma from individuals with long-term exposure to malaria in the Amazon area. The percent inhibition was calculated as 100 x (Rc—Rt)/Rc, where Rc is the average number of rosettes in the control wells, and Rt is the average number of rosettes in the test wells. Plasma samples with more than 50% of binding inhibition were considered positive.

### Prediction of DBPII—HLA class II binding affinity

To predict HLA-DR/-DQ binding affinities (IC50) we used the *P*. *vivax* DBP sequence (XP_001608387.1) from the NCBI database. Each potential 15-mer sequence frame was scored using the NetMHCIIpan-3.1 server (http://www.cbs.dtu.dk/services/NetMHCIIpan-3.1/), an improved version of the tool that permits a much more accurate binding core identification [28]. Binding affinity was given as the log IC50 value in nanomolar (nM), and the defined thresholds for strong and weak binders were <1.7 nM and <2.7 nM, respectively.

### Modelling the structural effects of the HLA class II variants

Homology models of the three DRB1 variants were generated using Modeller and Macro Model (Schrodinger, New York, NY) using an ensemble of previously solved X-ray crystal structures of the HLA II beta chain (PDB IDS: 1IEB Chain D, 3LQZ Chain B and 1SEB Chain B; 76%, 67% and 90% sequence identity respectively). The alpha chain was modelled using an ensemble of available X-ray crystal structures including PDB IDs: 2Q6W and 4AEN (Chain D and A respectively, 100% sequence identity). As previously described [29, 30], the models were then minimized using the MMF94s forcefield in Sybyl-X 2.1.1 (Certara L.P., St Louis, MO), with the final structure having more than 95% of residues in the allowed region of a Ramachandran plot. The quality of the models was confirmed with Verify3D. The models of the HLA II complex of the alpha and beta chains were built using X-ray crystal structures of the complex (PDB ID: 1IEB, 3LQZ, 1SEB, 2Q6W, 4AEN) to guide protein docking [31]. Two representative DBPII antigenic peptides (H1: FHRDITFRKLYLKRKL; H3: DEKAQQRRKQWWNESK) were modelled into each HLA II complex variant using the available crystal structures of the HLA II complexes to guide docking. Binding affinities were predicted using CSM-lig [32]. Model structures were examined using Pymol.

The structural consequences of each amino acid difference between the DRB1 variants were analyzed to account for all the potential effects of the mutations [33]. The effects of the variations on the stability of DRB1 and the HLA II complex were predicted using DUET [34], an integrated computational approach that optimizes the prediction of two complementary methods (mCSM-Stability and SDM). The effect of the differences on the protein-protein binding affinity between the alpha and beta chains to form the HLA II complex were predicted using mCSM-PPI [35]. The effect of the changes on the binding affinity of the HLA II complex for a model peptide were also analysed using mCSM-PPI, as previously described [36], mCSM-lig [37], and mCSM-AB [38]. These computational approaches represent the wild-type residues structural and chemical environment of a residue as a graph-based signature in order to quantitatively determine the change upon mutation in Gibb’s Free Energy of stability or binding.

### Statistical analysis

A database was created using Epidata software (http://www.epidata.dk). Linear correlation between two variables was determined by using the Spearman’s correlation coefficient. Differences in proportions were evaluated by chi-square (Χ2) test and, differences in medians were tested using either the Mann-Whitney or Kruskal–Wallis tests, with Dunn’s post hoc test, as appropriate. For allelic group comparison, differences in proportion were performed by Z-test or chi-square tests, or Fisher’s exact tests, as appropriate. Alleles frequencies for each locus (*DRB1*, *DQA1*, and *DQB1*) were summarized descriptively using frequencies and percentage for immunological categorical variables.

Overall associations with immunological responses and alleles from each locus of HLA class II were evaluated by comparing the allele frequencies between seronegative individuals and seropositive individuals from the baseline study using standard contingency tables. Based on the humoral immune response to DBPII from the three cross-sectional surveys, the long-term immune responses against DBPII were grouped into three categories: (i) *Persistent non-responder* (PNR)—absence of antibodies against DBPII in all three cross-sectional surveys; (ii) *transient responder* (TR)—antibodies detected in at least one cross-sectional survey; (iii) *Persistent responder* (PR)—individuals with detectable DBPII antibodies in all three cross-sectional studies. The association between HLA class II alleles (or haplotypes) and long-term immune response (PR or PNR groups) was analyzed by standard contingency tables (Chi-square and Fisher’s exact test, as appropriate), with two observations per subject (one for each allele). Alleles with a frequency of less than 0.01 were not included in the analysis.

Additionally, multiple logistic regression models with stepwise backward deletion were built to describe independent associations between covariates and HLA class II alleles or haplotypes and antibodies to DBPII. Covariates were selected for inclusion in the logistic models if they were associated with the outcome at the 15% level of significance in exploratory unadjusted analysis. Logistic regression models included the following covariates: age, gender, exposure to malaria (time of residence in the endemic area), self-reported malaria episodes, recent malaria infection and household location within the study area. Multivariate logistic regression was performed using Stata software version 10 (Stata Corporation, College Station, TX). Only variables associated with statistical significance at the 5% level were maintained in the final models. To avoid type II errors due to overly severe correction, statistical adjustment for multiple tests were not used [39, 40]. Type I errors were reduced by using multiple logistic regression models with stepwise backward deletion. Estimated genotype distribution between the observed and expected allelic frequencies was tested using the method described by Guo and Thompson [41] to verify Hardy-Weinberg equilibrium. Because the gametic phase was unknown, maximum-likelihood estimates of haplotype frequencies were obtained from multilocus genotype data and computed using the expectation-maximization (EM) algorithm [42]. Both procedures were performed using Arlequin software version 3.5 (http://cmpg.unibe.ch/software/arlequin35/) [43].

## Results

### Malaria infection and DBPII-specific antibodies at enrollment

We evaluated DBPII antibody responses in 336 unrelated subjects with a median of age 41 years and a 1.3:1 male-female ratio (Table 1). Age was significantly associated with a subject’s time of malaria exposure in the Amazon area (r = 0.82; p<0.0001, Spearman’s correlation test). At the time of the first blood collection, the overall prevalence of malaria was 5%, with 14 out of the 17 (82%) infections caused by *P*. *vivax* and 3 (18%) by *P*. *falciparum*. No *P*. *malariae* or mixed *Plasmodium* infections were diagnosed by either microscopy or Real-Time PCR. The 336 participants were followed up for an average of 7.5 months (10 days to 12 months), thus representing 2,514 person-months of follow-up. Based on parasitological-confirmed cases, the incidence rates of *P*. *vivax* malaria were 1.03 episodes per 100 person-months (95% confidence interval [CI] of 0.69–1.49/100 person-months) and 0.19 per 100 person-months for *P*. *falciparum* (95% CI of 0.03–0.32/100 person-month).

**Table 1:** Demographical, epidemiological and immunological data of the study population, 336 unrelated inhabitants of the agricultural settlement of Rio Pardo, Amazonas, Brazil.

Characteristic	
Age, median, years (IQR) ^a^	41 (25–51)
Gender ratio, male:female	1.3:1
Years of malaria exposure, median (IQR)^b^	33 (21–48)
Years of residence in Rio Pardo, median (IQR) ^c^	7.5 (4–12)
Previous malaria episodes, median (IQR)	5 (1–11)
Acute malaria infection, n (%) ^d^	17 (5.0)
* P*. *vivax*	14 (4.1)
* P*. *falciparum*	3 (0.9)
DBPII antibody response, positive (%) ^e^	122 (36)
DBPII binding inhibitory antibodies [n = 164], positive (%)^f^	58 (35.4)

^a^ IQR = interquartile range

^b^ Time living in the endemic area (Brazilian Amazon)

^c^ Time living in the agricultural settlement area

^d^ Malaria infection was detected by conventional light microscopy and/or real-time PCR at the time of their first blood-collection (individual baseline); no mixed infections were found in this study

^e^ ELISA-detected IgG antibodies targeting DBPII; results are expressed as the number (%) of individuals with a positive antibody response at the time of their first blood-collection

^f^ Functional assays with transfected COS cells to detect DBPII binding inhibitory antibodies (BIAbs) were performed on a subpopulation of the study sample

One hundred and twenty-two (36%) of the individuals enrolled in the study had ELISA-detected IgG antibodies to the main variant of DBPII circulating in the study area (Sal-1) (Table 1). Because not all DBPII IgG antibodies are able to block the interaction between the ligand (DBPII) and its receptor on the RBC surface (DARC), we evaluated the functional properties of the anti-DBPII antibodies. Due to the methodological constraints of performing functional assays, measurement of DBPII binding inhibitory antibodies (BIAbs) was performed on a representative subset of the study population comprising 164 individuals, matched for age, sex, malaria exposure and DARC alleles; 58 (35.4%) of these individuals showed BIAbs response against a predominant DBPII variant circulating in the study area (Table 1).

### Evaluation of long-term natural DBPII-specific IgG antibodies

**Fig 2:**
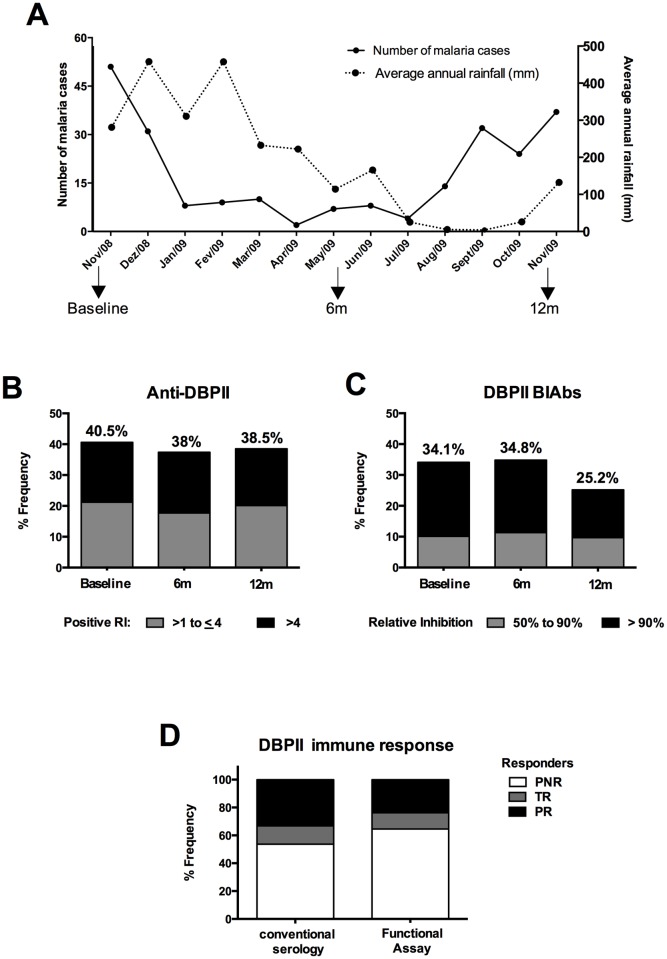
Temporal distribution of *P*. *vivax* malaria episodes and immune response targeting region II of the Duffy binding protein (DBPII), Rio Pardo Settlement, Amazonas, Brazil (Nov. 2008 –Nov. 2009). (**A)** Monthly malaria notifications in the Rio Pardo settlement (SVS/MS, Surveillance Service database, Brazilian Ministry of Health). Episodes of malaria, as determined by conventional microscopy, varied according to rainfall and season. Arrows indicate the time of the cross-sectional surveys (November, 2008; June 2009; and November, 2009). (**B**) DBPII IgG ELISA-detected antibody (Anti-DBPII) response remained stable during the follow up period (Baseline, and at 6 or 12 months). Results are expressed as a frequency (%) of responders, and a Reactivity Index (RI) of greater than 1.0 was considered positive. (**C**) DBPII binding inhibitory antibodies (BIAbs) had a slightly decreased frequency at the 12 months follow-up examination. BIAbs were evaluated by COS-7 cytoadherence assay, using plasma samples diluted to 1:40 (n = 164). Positive results were considered as DBPII–DARC binding inhibition of ≥50%. (**D**) Based on conventional (ELISA-detected IgG) and functional serology assays (DBPII BIAbs) at 12 months of follow-up, individuals were classified as *persistent non-responder* (PNR)—no antibody response detected any time-point of the follow-up; *transient responder* (TR)—antibodies detected in at least one cross-sectional survey; or *persistent responder* (PR)–antibodies detected in all cross-sectional surveys.

Since the number of malaria cases varied during the course of the study (Fig 2A), we evaluated the long-term antibody response at different levels of malaria transmission. Over three cross-sectional surveys, at 6-month intervals, between 38 to 40% of individuals developed DBPII IgG antibodies, as detected by conventional serology (Fig 2B). Considering the inhibitory antibody response, there was a slight decrease in the frequency of BIAbs at the time of the 3rd cross-sectional survey (34–35% to 25%) (Fig 2C). Finally, the 12-month follow-up study allowed individuals to be classified as persistent non-responders (PNR), transient responders (TR), or persistent responders (PR) for either conventional serology or BIAbs immune response (Fig 2D). For conventional and inhibitory antibody responses, the frequency of acute malaria infections was similar between the PNR, TR or PR groups (p>0.05 for all comparisons).

### Distribution of HLA class II in the study population and association with ELISA-detected DBPII IgG antibodies

Of the HLA class II loci that were genotyped in the study population, we found 13 *HLA-DRB1*, 6 *HLA-DQA1*, and 5 *HLA-DQB1* allele groups. As expected, *HLA-DRB1* was the most polymorphic locus with 46 alleles identified; there were 21 and 13 *DQB1* and *DQA1* alleles, respectively. For each HLA class II locus, the predominant alleles (frequency ≥ 0.01) were listed in the S1 Fig.

**Fig 3:**
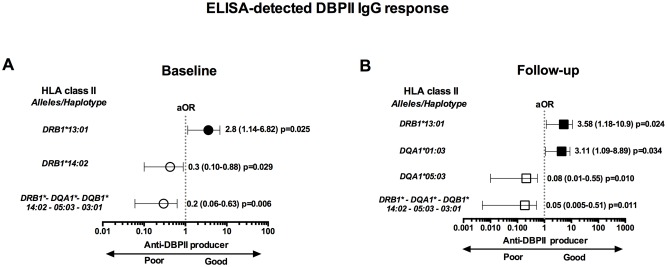
Association between HLA class II alleles (*DRB1*, *DQA1*, and *DQB1* loci) and *Plasmodium vivax* Duffy binding protein (DBPII) antibody responses in individuals naturally exposed to malaria. (**A**) At enrollment (baseline), adjusted Odds Ratio (aOR) analysis for the association of HLA class II (alleles and haplotypes) and the presence (*black circle)* or absence (*white circle*) of DBPII IgG antibody (Ab) response, as detected by ELISA. (**B**) After 12-month follow-up, adjusted Odds Ratio (aOR) analysis for the association of HLA class II (alleles and haplotypes) and the long-term DBPII antibody response; individuals were classified as persistent non-responder (PNR, *white square*) or responder (PR, *black square*), as described in legend of Fig 2. Adjusted ORs analyses were performed using multivariable logistic regression models adjusted for confounding variables (age, sex, previous malaria episodes, and dwelling location). Variables associated with DBPII antibody at 5% level (p < 0.05) were maintained in the final models.

In a preliminary analysis, the effect of HLA class II genes on conventional DBPII antibody response was evaluated at the time of the first blood collection (S1 Table). While three HLA class II alleles (DRB1*13:*01*, DQA1*01:*03*, DQB1*06:*03*) were positively associated with anti-DBPII antibody response, six alleles (DRB1*10:*01*, DRB1*14:*02*, DQA1*01:*01*, DQA1*05:*03*, DQB1*03:*01*, DQB1*05:*01*) were negatively associated. Nevertheless, using multiple logistic regression models, only the DRB1*13:*01* (presence) and DRB1*14:*02* (absence) alleles were significant predictors of anti-DBPII antibodies (Fig 3A).

Since combinations of HLA alleles are inherited together in the genome more often than expected, we further evaluated the association between ELISA-detected DBPII-specific antibodies and HLA class II haplotypes. In total, 126 combinations of specific *DRB1*, *DQA1*, *DQB1* haplotypes were found, and for 27 of them (frequency ≥ 0.01) it was possible to estimate the individual probability of developing DBPII antibodies. Adjusted logistic regression analysis identified a single haplotype associated with poor production of DBPII antibodies, with individuals carrying the haplotype DRB1*14:02-DQA1*05:03-DQB1*03:*01* 5-times less likely to develop a conventional DBPII antibody response (Fig 3A). In addition, for each HLA class II locus analyzed (*DRB1*, *DQA1*, and *DQB1*), the genotype frequencies were confirmed to be in Hardy-Weinberg equilibrium (for the responder vs. non-responder groups).

Next, we investigated whether the status of persistent responder (PR) or non-responder (PNR) was HLA class II-linked at the time of the 12-month follow-up collection. The frequencies of some HLA class II alleles were significantly different between the PR and PNR groups (S2 Table). An adjusted odds ratio analysis confirmed that two alleles were associated with the status of long-term responder, and a single allele was associated with the absence of an antibody response (Fig 3B). More specifically, while individuals carrying either the DRB1*13:*01* or DQA1*01:*03* alleles had an increased probability of a sustained antibody response, the DQA1*05:*03* allele carriers were associated with the status of persistent non-responders (Fig 3B). It is noteworthy that the DQA1*05:*03* allele aggregated in a specific haplotype (DRB1*14:02-DQA1*05:03-DQB1*03:*01)* that was primarily associated with the absence of an antibody response (Fig 3A), and this haplotype was in strong linkage disequilibrium (Δ = 1.0; P = 0).

### HLA class II variability and DBPII inhibitory immune response

**Fig 4:**
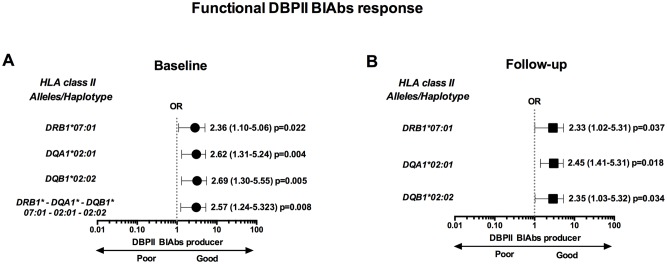
Association between HLA class II alleles (*DRB1*, *DQA1*, and *DQB1* loci) and *Plasmodium vivax* Duffy binding protein (DBPII) binding Inhibitory Antibodies (BIAbs) in individuals naturally exposed to malaria. (**A**) At enrollment (baseline), Odds Ratio (OR) analyses for the association of HLA class II (alleles and haplotypes) and DBPII BIAbs response, as detected by functional assays with DBPII- transfected COS cells; *black circles* represent alleles and haplotypes of HLA class II associated with a positive BIABs response. (**B**) After 12-month follow-up, Odds Ratio analyses for the association of HLA class II (alleles and haplotypes) and long-term DBPII BIAbs response; individuals were classified as persistent non-responder (PNR) or responder (PR, *black square*), as described in legend of Fig 2. Odds Ratio analyses realized using standard contingency tables (p < 0.05; qui-square and Fisher’s exact test, as appropriate).

Further experiments investigated whether HLA class II polymorphisms interfered with the functional proprieties of DBPII antibodies. The three cross-sectional measures of DBPII BIAbs responses were performed on a subset of the study population comprising 164 individuals (Table 1), with responders (n = 58) and non-responders (n = 106) matched for age, sex, and malaria exposure. Three alleles (DRB1*07:*01*, DQA1*02:*01*, and DQB1*02:*02*) were overrepresented in DBPII BIAbs responders (S3 Table), and these same alleles aggregated in a haplotype (Fig 4A), which was in linkage disequilibrium (Δ = 0.90 and 0.94, for responders and non-responders, respectively). Interestingly, the long-term persistence of anti-DBPII responses (determined at the 12 month follow-up analysis) was also associated with those same three HLA class II alleles (Fig 4B). Unfortunately, the small size of the sample precluded use of adjusted odds ratio analyses. Nonetheless, responder and non-responder groups were matched by the confounding variables (age, sex, malaria exposure, and dwelling localization).

### *In silico* binding prediction of DBPII peptides to class II allelic variants

Since persistence and functional properties of DBPII antibodies were influenced by class II allelic variants, we investigated whether differences in the affinity of DBPII peptides for class II molecules might contribute to the observed difference in responses. Based on predicted binding affinity between DBPII peptides and HLA-DR/DQ alleles, we found unexpected differences in affinity in favor of the non-responder allele carriers. Actually, the HLA-DR allele linked to non-responders (DRB1*14:*02)* appeared to have a higher binding affinity for the peptides (low IC50 values) than the HLA–DR alleles that were associated with responders (DRB1*07:*01* and DRB1*13:*01)* (S2 Fig). In spite of that, different HLA-DR binding profiles were found for previously identified DBPII epitopes [44–47]. Of note, the recently described broadly neutralizing DBPII epitopes (2D10/2H2 and 2C6) had low binding affinity for all of the Class II molecules analyzed (S2B Fig). Considering HLA-DQ, the model also showed a higher predicted binding affinity for HLA-DQA1/B1 molecules linked to non-responders.

### Structural analysis of HLA-DRB1 variants

**Fig 5:**
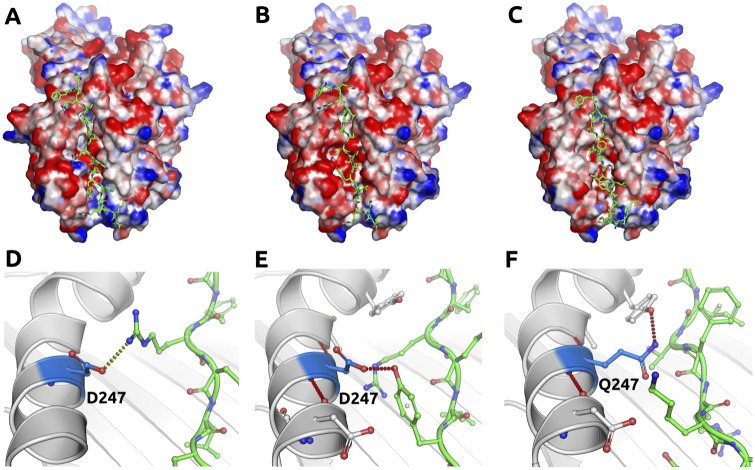
The HLA II complex peptide-binding groove. The figure presents a representation of the HLA II complex electrostatic surface potential, and interactions made by residue 99 of DRB1 for the responder alleles DRB1*07:*01* (A and D, respectively) and DRB1*13:*01* (B and E, respectively), and the non-responder allele DRB1*14:*02* (C and F, respectively). The DBPII H1 peptide is shown inside the binding groove as green sticks. Hydrogen bonds are shown as red dashed lines.

Structural analysis of the three HLA-DRB1 variants revealed a number of interesting differences in the peptide-binding groove (S3 Fig), with significant alterations to its electrostatic potential, and reduced affinity for the model peptides (Fig 5A–5C), which we propose as an explanation for the differences in subject responses. The antigenic surface of DBPII has a strong positive charge (S4 Fig), which is suggestive of a binding preference for antibodies targeting positively charged epitopes such as those that will be preferentially bound by the DRB1*07:*01* and DRB1*13:*01* variants. Interestingly, while the DRB1*14:*02* and DRB1*13:*01* variants are the closest in sequence identity, the docked peptides were most similar between the two responder variants (rmsd of the peptides < 3.8 Å), whilst the non-responder did not dock in a similar way (rmsd of the peptides > 8 Å). This supports the suggestion that the non-responder variant leads to reduced antigen presentation.

Overall, while the non-responder-associated variant (DRB1*14:*02*) shares 81.2% and 95.5% sequence identity to DRB1*07:*01* and DRB1*13:*01*, respectively, the sequence identity is lower in the groove region within 5 Å of the presented antigen (DRB1*07:*01*–73.3%; DRB1*13:*01*–89.6%). While the majority of the differences between the variants are located within the peptide-binding domain, this change in the nature of the antigen-binding groove is evident in differences between their isoelectric points, with DRB1*07:*01* and DRB1*13:*01* having slightly acidic pI’s (6.5 and 7.0 respectively), and DRB1*14:*02* being basic (7.7). It was also reflected in the energy calculations, with DRB1*14:*02* having an overall Coulomb energy approximately 1.5% lower than either of the responder variants.

One significant difference between the good and poor responder variants was the presence of a glutamine residue at position 99 (247 according to the Protein Data Bank, PDB) in DRB1*14:*02*, compared to aspartic acid in DRB1*07:*01* and DRB1*13:*01*. This residue side-chain points into the peptide-binding groove and is located approximately 5 Å from the antigen peptide backbone, making a key hydrogen bond (Fig 5D and 5E). Here, loss of the acidic residue in the peptide-binding groove was predicted to reduce the H1 and H3 peptide binding affinity (ΔΔG < -0.8 kCal/mol), and is likely to lead to altered antigen binding and presentation profiles, and hence the poor response seen for DRB1*14:*02* variant carriers.

## Discussion

The HLA molecules encoded by MHC class II genes are responsible for presenting peptide epitopes to CD4+ T helper cells. Consequently, it is reasonable to postulate that polymorphism in the HLA class II region may account for the variation in DBPII antibody responses. Unfortunately, most antibody prevalence data on malaria have been collected by cross-sectional analysis at a single time point, which might led to misclassification of individual immune responsiveness. Therefore, in this study we conducted a longitudinal study, collecting serum from the same individuals over a period of 12 months, to obtain a reliable estimate of DBPII antibody prevalence. The genetic profile of HLA class II in the study population was similar to other populations of the Brazilian Amazon [48], which are characterized by an interethnic admixture with high proportions of European and Amerindian groups [49]. Accordingly, in the study population 44% had European ancestry, followed by 38% Amerindian, and 18% African ancestry. For other Brazilian regions, the contribution of European ancestry has ranged from 40% in the Northeast to >70% in the Southeast and South [50, 51]. As expected, the Native-American ancestry in the study area (38%) was representative of the Amazonian region, where Amerindian ancestry is much higher that in other Brazilian regions (<10%) [50].

Our current study confirms the heritability of antibody responses to DBPII, with genetic variation in HLA class II molecules influencing both the development and persistence of an individual’s anti-DBPII IgG antibody response. Accordingly, multivariate analyses adjusted for potential confounding variables showed effects of alleles linked to the DR and DQ loci on the presence (DRB1*13:*01*) and persistence (DRB1*13:*01* and DQA1*01:*03*) of ELISA-detected DBPII IgG antibodies. On the other hand, two alleles were associated with DBPII-non-responsiveness, DRB1*14:*02* and DQA1*05:*03*, and these comprised a single haplotype (DRB1*14:02-DQA1*05:03-DQB1*03:*01)* that significantly reduced the development of anti-DBPII IgG at any time during the follow-up study (baseline, 6 and 12 months later). Interestingly, the alleles in the aforementioned haplotype were in strong linkage disequilibrium, which demonstrated that these poor-responder alleles are inherited together more often than expected by chance. So far, only a single study has investigated the association between HLA and DBPII antibodies [52]. Although in that case the authors were unable to demonstrate an association between HLA type and ELISA-detected DBP IgG antibodies, and the relatively limited number of responders did not allow any final conclusions about the highly polymorphic HLA class II and DBP antibodies.

Here, the assessment of long-term antibody response was essential to strengthen the conclusion that there was an increased susceptibility of DRB1*13:*01* carriers to develop and sustain their anti-DBPII IgG antibody response. Furthermore, these data confirmed that individuals harboring the haplotype DRB1*14:02-DQA1*05:03-DQB1*03:*01* were persistent non-responders. Due to the overall scarcity of data combining analysis of HLA and immune responses to *P*. *vivax*, further confirmation of these associations in other malaria endemic areas is needed. Despite the remarkable lack of data on this subject, systematic review and meta-analysis studies have identified a link between the DRB1*13:*01* allele and increased antibody responses to vaccines for other microbial infections, including hepatitis B, influenza virus, serogroup C meningococcus, and MMR-II (measles and rubella virus) [53, 54]. Additionally, the DRB1*14 allelic group has been associated with a poor humoral response to HBsAg vaccination [55].

Many field studies examining the immune response to malaria have focused on measuring the concentrations of antibodies to vaccine candidate antigens, while less attention has been paid to complementary approaches defining the functional relevance of these antibodies. By using an *in vitro* assay to quantify inhibition of DBPII–erythrocyte binding [9, 56], we demonstrated that DBPII binding inhibitory antibodies (BIABs) were associated with three alleles (DRB1*07:*01*, DQA1*02:*01* and DQB1*02:*02*), which are in linkage disequilibrium and were found to be part of a single haplotype. Notably, these three alleles were associated with the presence of BIAbs antibodies and were also associated with the persistence of this inhibitory response. Therefore, our observations may explain previous results showing that the majority of people who are naturally exposed to *P*. *vivax* do not develop antibodies that inhibit the DBPII-DARC interaction, but once they are acquired these BIAbs seem to be stable under continuous exposure to malaria transmission [11, 13].

Intriguingly, a significant number of pharmacogenetic studies have identified HLA-DRB1*07:*01* carriers (in less extension, DQA1*02:*01* and DQB1*02:*02)* as being more susceptible to side effects of biological therapy due to the activation of immune response drug-induced [57–59]. Notably, part of the side effect could be explained by the higher production of neutralizing antibodies against drugs (or their metabolites) in the HLA-DRB1*07:*01* and/or DQA1*02 carriers [59, 60]. Although drug-specific antibodies are undesirable in therapies involving biological proteins, these findings reinforce our results of a much higher frequency and persistence of DBPII neutralizing antibodies in individuals harboring those alleles HLA class II. In future studies, functional analysis of a greater number of individuals might allow for more robust statistical comparisons.

It is noteworthy that the class II alleles associated with DBPII inhibitory activity were not associated with the conventional IgG antibody responses. Likewise, alleles (or haplotypes) associated with ELISA-detected IgG antibodies were not associated with DBPII BIAbs. These results are not completely unexpected because quantitative receptor binding assays distinguish between antibodies that recognize DBPII and those that inhibit binding to DARC receptor. Here, the DBPII/DARC interaction was assessed by using an established cytoadherence assay based upon multivalent interactions between DBPII on the surface of COS-7 cells and DARC expressed in RBCs [56]. As a consequence, we and others have demonstrated a moderate correlation between DBPII BIAbs and ELISA anti-DBPII antibodies (revised in [8]). Overall, our results emphasize the relevance of examining functional aspects of the immune response, particularly in the case of immunogens such as DBPII, in which the goal of vaccination would be to enhance broadly neutralizing antibodies targeting invasion-blocking epitopes.

To gain insights into the difference between good and poor HLA responders, we sought to investigate whether natural HLA-DR/DQ allelic differences could be explained with respect to binding affinity of DBPII epitopes. While predicted DBPII epitopes have a unexpected moderate-to-high affinity for non-responder alleles, the binding affinity of previously described DBPII epitopes [44–47] was much more variable, including low binding affinities of recently described DBPII B-cells epitopes associated with strain-transcending immunity [47]. However, it seems inappropriate to extrapolate our findings to conformational B cell epitopes because the prediction analyses used here were largely determined by the primary amino acid sequence of the peptide-binding core. In this context, the development of tools for reliably predicting B-cell epitopes, particularly for predicting conformational epitopes, remains a major challenge in immunoinformatics [61].

Although the predicting peptide-HLA binding affinity method used here (NetMHCIIpan—www.cbs.dtu.dk/services/NetMHCIIpan-3.1) [62] seems to be a suitable predictive algorithm for T cell epitopes [28], we performed a further detailed structural in silico analysis of the HLA-DRB1 variants. Significantly, the majority of the differences between HLA-DRB1 variants (good vs. poor responders) were located within the peptide-binding domain, leading to significant changes in the nature of the antigen-binding groove. A striking structural difference between HLA-DRB1 variants was the presence of a glutamine residue at position 99 in the poor responder allele (DRB1*14:02), as compared to aspartic acid in the good responder alleles (DRB1*07:01 and DRB1*13:01). Remarkably, mutation of the corresponding residue has previously been shown to result in loss of the ability of HLA-DP2 to present the metal beryllium to T cells, in genetically susceptible to chronic beryllium- disease [63]. Notably, this single mutation seems to drive helper CD4 T cells in susceptible individuals to secrete Th1-type cytokines, such as gamma-interferon, but not IL-4, leading to beryllium-induced hypersensitivity and chronic beryllium-disease [64, 65]. Consequently, we speculate that the mutation found here in the peptide-binding groove (D247 vs. Q247) is likely to change the outcome of the CD4+T cells immune response. Accordingly, the loss of the acidic residue in the peptide binding groove was predicted to reduce DBPII-specific peptide binding affinity (H1 and H3), which is expected to lead to altered antigen binding and presentation profiles, and hence poor response of carriers of the DRB1*14:02 variant. It strengthens the findings that DRB1*14:02 could be more frequently involved with a poor antibody production [55], while the DRB1*13:01 allele produces a much more robust antibody response [53, 54]. Future studies are required to determine differences in the functionalities of DBPII epitopes in the context of different HLA molecules.

Notwithstanding the relevance of our results, the current study has some limitations. As we focused on the highly variable HLA class II genes it may not have been possible to discriminate between causal alleles and variation that is due to the linkage disequilibrium (LD) between alleles. In fact, in most association studies it has been difficult to pinpoint the causal variants within this genetic complex due to strong LD, population heterogeneity, and the high density of immune-related genes [66]. Such studies have proven most successful for diseases with one prominent predisposing genetic factor mapping to either the class I or class II region [67]. In addition, the associations described in the present study are most likely multifactorial, and depend on several additional factors related to the parasite and host environment. Although the structural analysis of DRB1 variants described here suggested that specific alleles might influence anti-DBPII antibody responses, these results indicate a first step towards the understanding of DBPII immune response in the context of different HLA class-II variants. We are confident that future cellular assays can be pursued to confirm and identify mechanisms associated with good and poor antibody responders. Finally, knowledge of the relative binding affinities of DBPII peptides for class II molecules associated with good and poor responses to this major *P*. *vivax* blood-stage vaccine candidate might lead to strategies for controlling the type of helper T cells activated in response to DBPII.

## Supporting Information

S1 Fig Alleles frequencies of HLA class II (*DRB1*, *DQA1* and *DQB1* loci) of the study population (n = 336), Amazonas state, Brazil. Most frequent alleles of HLA class II (**A**) *DRB1*; (**B**) *DQA1*; and (**C**) *DQB1*. Allele frequencies greater than or equal to 0.01 were included in the figure. For each locus, lowercase letters (a-g) indicate statistically significant differences between allele groups (p<0.05, Z-test).
(TIF)

S2 Fig Binding prediction of *P*. *vivax* DBP peptides to MHC class II molecules. Binding affinity is depicted for the whole molecule (A) and for the Duffy Binding-Like domain (region II). (B) Binding affinity is given as the log of the IC50 value (nM) for good (*blue line*) and poor (*black line*) DBPII responders. The predictions for good responders represent the average of IC50 values of the DRB1*07:*01* and DRB1*13:*01* alleles; values for poor responders refer to the prediction for the DRB1*14:*02* allele. The horizontal lines indicate the threshold for defining strong (<1.7) and weak binders (from 1.7 to 2.7) based on predicted affinity. Predicted values of IC50 were specified in a sliding window of 15 amino acids in length. Previously identified epitopes in region II of PvDBP were indicated [44–47]. Boundaries of regions I-VI of the protein were defined as previously described [68]. The three subdomains (SD) in the region are indicated by colored bars: SD1 (green), SD2 (blue) and SD3 (orange) [69].
(TIF)

S3 Fig Differences in peptide binding groove between HLA class II variants. The DRB1*14:*02* allele is depicted in yellow, DRB1*07:*01* in green (A) and DRB1*13:*01* in blue (B). The peptide is shown as a cartoon in yellow. Residues within 5 Å of peptide for the three variants are shown as sticks. Residues in pink differ between variants.
(TIF)

S4 Fig DBPII surface electrostatics. This figure presents a representation of the DBPII electrostatic surface potential.
(TIF)

S1 Table Association between antibody responses against *P*. *vivax* Duffy binding protein (DBPII) and HLA class II (*DRB1*, *DQA1*, and *DQB1*) alleles of individuals naturally exposed to malaria. (PDF)

S2 Table Association between the long-term Duffy binding protein (DBPII) antibody response and HLA class II (*DRB1*, *DQB1* and *DQA1)* alleles of individuals naturally exposed to malaria. (PDF)

S3 Table Association between binding inhibitory antibody (BIAbs) response against *P*. *vivax* Duffy binding HLA class II (*DRB1*, *DQB1* and *DQA1*) exposed to malaria. (PDF)

S1 File Data file of HLA class II and antibody immune response. (XLSX)
