# Supplementary figures and images for: The Presence, Persistence and Functional Properties of Plasmodium vivax Duffy Binding Protein II Antibodies Are Influenced by HLA Class II Allelic Variants
Source: PLoS Negl Trop Dis. 2016 Dec 13;10(12):e0005177. doi: 10.1371/journal.pntd.0005177 (PMC5154503; doi:10.1371/journal.pntd.0005177)

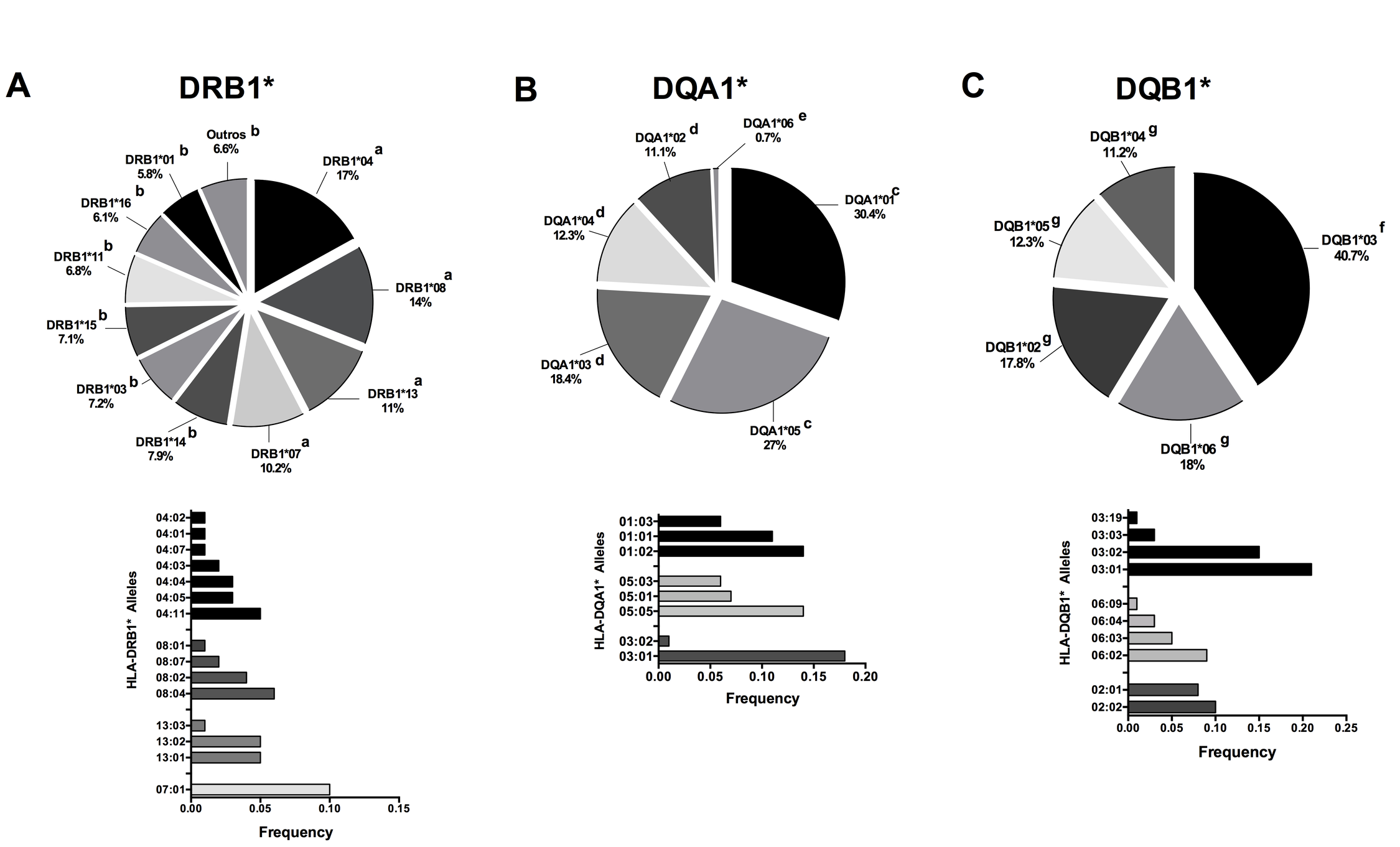

Supplement: S1 Fig — Most frequent alleles of HLA class II (A) DRB1; (B) DQA1; and (C) DQB1. Allele frequencies greater than or equal to 0.01 were included in the figure. For each locus, lowercase letters (a-g) indicate statistically significant differences between allele groups (p<0.05, Z-test). (TIF) [file pntd.0005177.s001.tif]

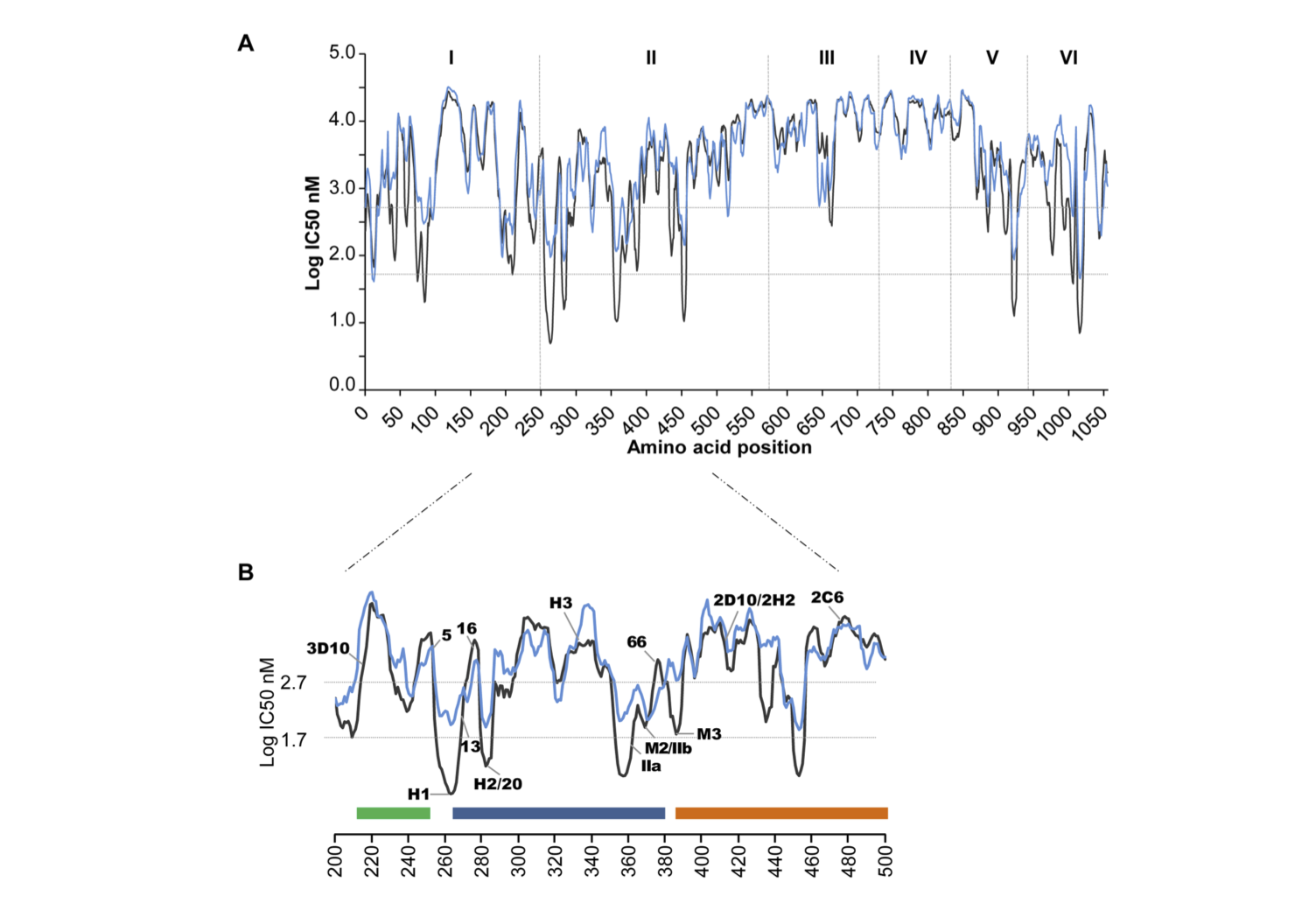

Supplement: S2 Fig — Binding affinity is depicted for the whole molecule (A) and for the Duffy Binding-Like domain (region II). (B) Binding affinity is given as the log of the IC50 value (nM) for good (blue line) and poor (black line) DBPII responders. The predictions for good responders represent the average of IC50 values of the DRB1*07:01 and DRB1*13:01 alleles; values for poor responders refer to the prediction for the DRB1*14:02 allele. The horizontal lines indicate the threshold for defining strong (<1.7) and weak binders (from 1.7 to 2.7) based on predicted affinity. Predicted values of IC50 were specified in a sliding window of 15 amino acids in length. Previously identified epitopes in region II of PvDBP were indicated [44–47]. Boundaries of regions I-VI of the protein were defined as previously described [68]. The three subdomains (SD) in the region are indicated by colored bars: SD1 (green), SD2 (blue) and SD3 (orange) [69]. (TIF) [file pntd.0005177.s002.tif]

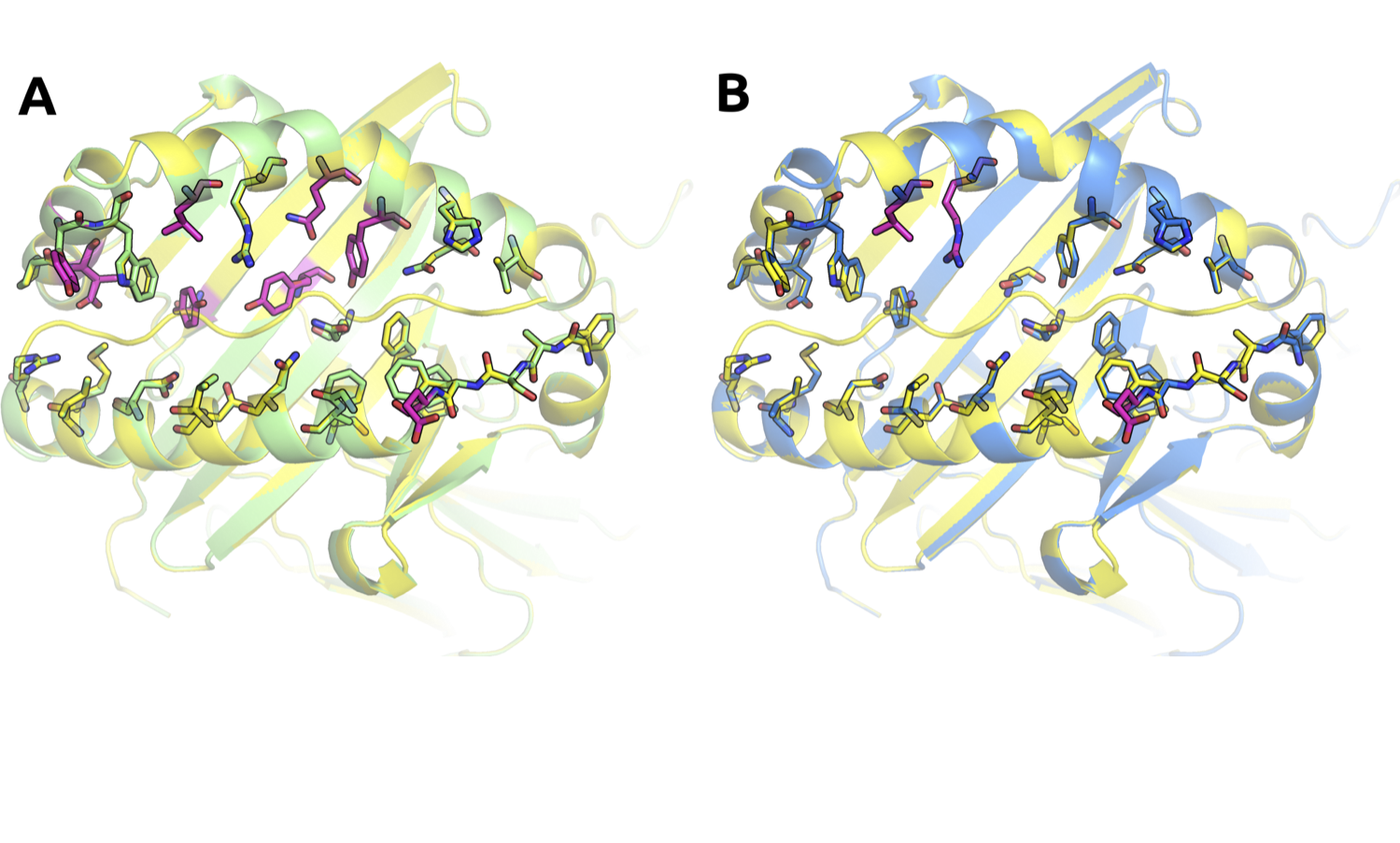

Supplement: S3 Fig — The DRB1*14:02 allele is depicted in yellow, DRB1*07:01 in green (A) and DRB1*13:01 in blue (B). The peptide is shown as a cartoon in yellow. Residues within 5 Å of peptide for the three variants are shown as sticks. Residues in pink differ between variants. (TIF) [file pntd.0005177.s003.tif]

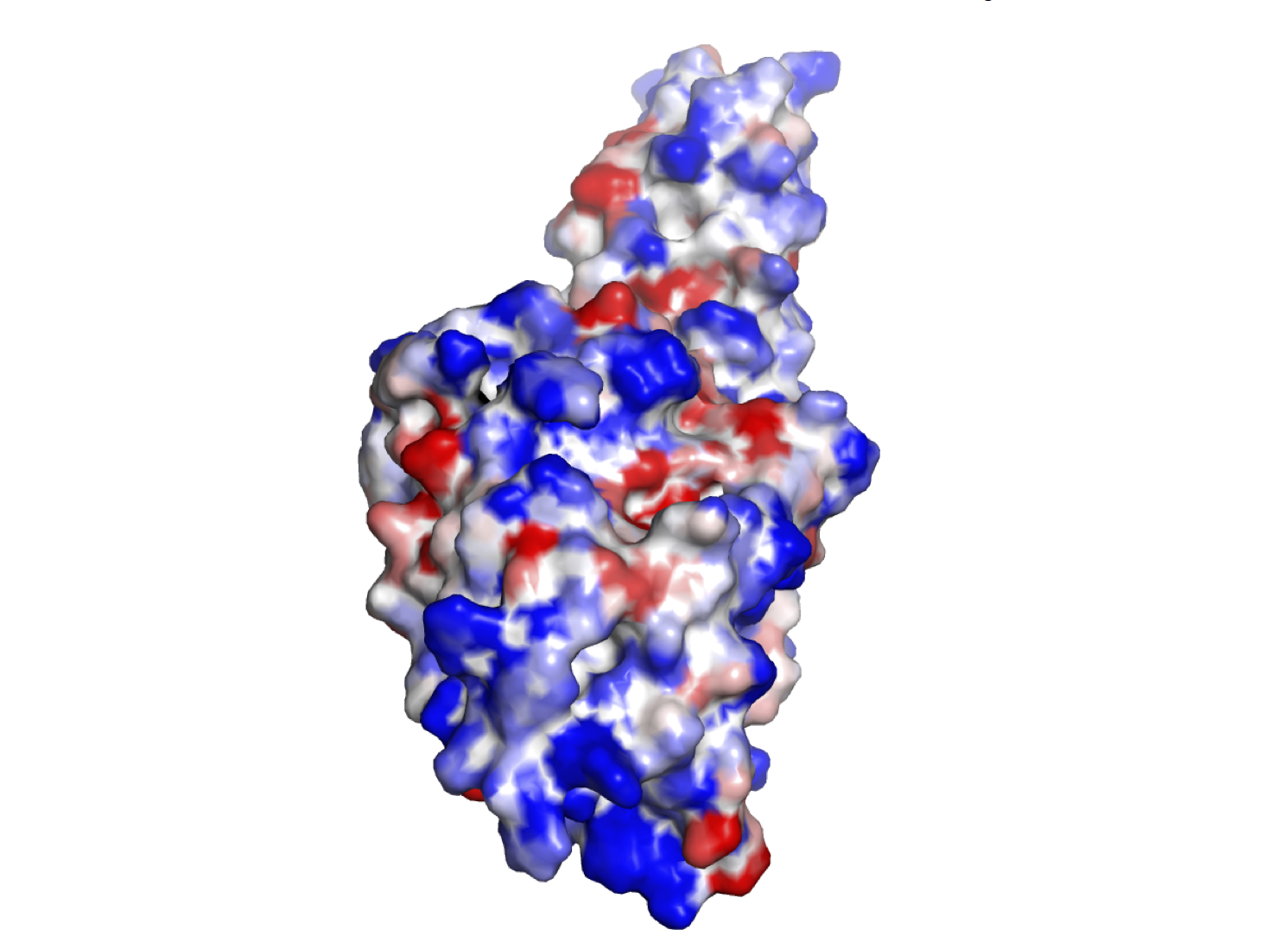

Supplement: S4 Fig — This figure presents a representation of the DBPII electrostatic surface potential. (TIF) [file pntd.0005177.s004.tif]
