# Supplementary material for: The Presence, Persistence and Functional Properties of Plasmodium vivax Duffy Binding Protein II Antibodies Are Influenced by HLA Class II Allelic Variants
Source: PLoS Negl Trop Dis. 2016 Dec 13;10(12):e0005177. doi: 10.1371/journal.pntd.0005177 (PMC5154503; doi:10.1371/journal.pntd.0005177)
Supplement: S1 Table — (PDF) [file pntd.0005177.s005.pdf]

S1 Table. Association between antibody responses against *P. vivax* Duffy binding protein (DBPII) and HLA class II (*DRB1, DQA1,* and *DQB1*) alleles of individuals naturally exposed to malaria

*HLA-DRB1**	DBPII Antibody response (ELISA)		OR (95% CI)	p-value
	Negative (N=181) n (%)	Positive (N=124) n (%)		
01:01	8 (2.2)	2 (0.8)	0.36 (0.08-1.71)	0.180
01:02	13 (3.6)	11 (4.5)	1.24 (0.55-2.82)	0.601
03:01	24 (6.7)	12 (4.9)	0.71 (0.35-1.46)	0.354
03:02	4 (1.1)	2 (0.8)	0.73 (0.13-4.00)	0.713
04:03	6 (1.7)	5 (2.0)	1.21 (0.37-4.04)	0.746
04:04	9 (2.5)	11 (4.5)	1.82 (0.74-4.46)	0.185
04:05	11 (3.1)	10 (4.0)	1.34 (0.56-3.20)	0.511
04:07	2 (0.6)	4 (1.6)	2.94 (0.53-16.3)	0.193
04:11	17 (4.7)	16 (6.5)	1.40 (0.69-2.82)	0.349
07:01	35 (9.7)	27 (10.9)	1.14 (0.67-1.94)	0.629
08:01	3 (0.8)	5 (2.0)	2.45 (0.58-10.4)	0.207
08:02	20 (5.6)	7 (2.8)	0.49 (0.20-1.19)	0.114
08:04	20 (5.6)	15 (6.1)	1.10 (0.55-2.19)	0.788
08:07	5 (1.4)	6 (2.4)	1.76 (0.53-5.86)	0.345
09:01	5 (1.4)	4 (1.6)	1.68 (0.31-4.40)	0.818
10:01	15 (4.2)	3 (1.2)	0.28 (0.08-0.99)	0.035[a]
11:01	11 (3.1)	14 (5.7)	1.90 (0.85-4.28)	0.112
11:04	3 (0.8)	7 (2.8)	3.47 (0.88-13.6)	0.057
12:01	10 (2.8)	2 (0.8)	0.29 (0.06-1.31)	0.087
13:01	13 (3.6)	19 (7.7)	2.22 (1.07-4.60)	0.027[a]
13:02	15 (4.2)	13 (5.3)	1.28 (0.60-2.73)	0.527
14:01	7 (1.9)	3 (1.2)	0.62 (0.16-2.42)	0.488
14:02	23 (6.4)	7 (2.8)	0.43 (0.18-1.00)	0.047[a]
14:06	5 (1.4)	2 (0.8)	0.58)-.11-3.01)	0.511
15:01	15 (4.2)	8 (3.2)	0.76 (0.32-1.84)	0.556
15:03	9 (2.5)	10 (4.0)	1.64 (0.66-4.11)	0.282
16:02	25 (6.9)	9 (3.6)	0.51(0.23-1.11)	0.083

*HLA-DQA1**	Negative (N=200) n (%)	Positive (N=136) n (%)	OR (95% CI)	p-value
01:01	52 (13.1)	22 (8.2)	0.59 (0.35-1.00)	0.049[a]

01:02	51 (12.8)	40 (14.9)	1.18 (0.76-1.85)	0.448
01:03	15 (3.8)	23 (8.6)	2.39 (1.21-4.68)	0.009[a]
02:01	41 (10.3)	33 (12.3)	1.21 (0.75-1.98)	0.428
03:01	63 (15.8)	56 (20.8)	1.40 (0.94-2.08)	0.099
04:01	48 (12.1)	33 (12.3)	1.01 (0.63-1.64)	0.936
05:01	33 (8.3)	13 (4.8)	0.56 (0.29-1.09)	0.084
05:03	32 (8.0)	10 (3.7)	0.44 (0.21-0.92)	0.024[a]
05:05	55 (13.8)	36 (13.4)	0.96 (0.61-1.51)	0.872

HLA-DQB1*	**Negative (N=200) n (%)**	**Positive (N=136) n (%)**	**OR (95% CI)**	**p-value**
02:01	33 (8.3)	20 (7.4)	0.88 (0.49-1.57)	0.669
02:02	34 (8.5)	32 (11.8)	1.43 (0.86-2.39)	0.164
03:01	96 (24.1)	47 (17.3)	0.66 (0.45-0.98)	0.036[a]
03:02	54 (13.6)	47 (17.3)	1.33 (0.87-2.04)	0.181
03:03	14 (3.5)	7 (2.6)	0.73 (0.29-1.83)	0.497
04:02	43 (10.8)	32 (11.8)	1.10 (0.68-1.80)	0.686
05:01	48 (12.1)	18 (6.6)	0.52 (0.29-0.91)	0.021[a]
05:03	6 (1.5)	2 (0.7)	0.49 (0.10-2.43)	0.369
06:02	36 (9.0)	24 (8.9)	0.98 (0.57-1.68)	0.933
06:03	12 (3.0)	19 (7.0)	2.42 (1.15-5.10)	0.016[a]
06:04	10 (2.5)	8 (3.0)	1.18 (0.46-3.03)	0.730
06:09	2 (0.5)	5 (1.8)	3.72 (0.71-19.4)	0.094

ELISA-detected IgG antibodies allow individual classification as positive (Reactivity index, RI > 1.0) or negative (RI <1.0). Only alleles with frequency higher or equal than 1% were analyzed; each individual corresponded to two observations (two HLA class-II alleles each). [a] Statistically significant differences (p<0.05, Qui-square test or Fisher's exact test as appropriate). [b] The distribution of allelic groups of each locus of HLA class II (*DRB1, DQA1*, and *DQB1*) are in equilibrium in this population, according to Hardy-Weinberg law (p<0.05).
