# Supplementary material for: The Presence, Persistence and Functional Properties of Plasmodium vivax Duffy Binding Protein II Antibodies Are Influenced by HLA Class II Allelic Variants
Source: PLoS Negl Trop Dis. 2016 Dec 13;10(12):e0005177. doi: 10.1371/journal.pntd.0005177 (PMC5154503; doi:10.1371/journal.pntd.0005177)
Supplement: S2 Table — (PDF) [file pntd.0005177.s006.pdf]

S2 Table. Association between the long-term Duffy binding protein (DBPII) antibody response and HLA class II (*DRB1, DQB1* and *DQA1)* alleles of individuals naturally exposed to malaria

*HLA-DRB1**	DBPII Antibody response		OR (95% CI)	p-value
	PNR (N=118) n (%)	PR (N=73) n (%)		
01:01	4 (1.7)	2 (1.1)	0.81 (0.14-4.50)	0.810
01:02	11 (4.7)	7 (4.0)	1.03 (0.39-2.74)	0.941
03:01	14 (5.9)	6 (3.4)	0.68 (0.25-1.82)	0.448
03:02	3 (1.3)	1 (0.6)	0.54 (0.06-5.25)	0.589
04:01	15 (6.4)	9 (5.1)	0.79 (0.34-1.86)	0.594
04:03	5 (2.1)	4 (2.3)	1.07 (0.28-4.06)	0.916
04:04	6 (2.5)	11 (6.3)	2.56 (0.93-7.05)	0.061
04:05	8 (3.4)	4 (2.3)	0.66 (0.20-2.24)	0.505
04:07	2 (0.8)	3 (1.7)	2.03 (0.33-12.3)	0.432
04:11	13 (5.5)	10 (5.7)	1.03 (0.44-2.41)	0.940
07:01	21 (8.9)	16 (9.1)	1.02 (0.52-2.02)	0.946
08:01	2 (0.8)	3 (1.7)	2.03 (0.33-12.3)	0.432
08:02	13 (5.5)	5 (2.9)	0.50 (0.18-1.43)	0.190
08:04	12 (5.1)	4 (2.3)	0.43 (0.14-1.37)	0.144
08:07	4 (1.7)	3 (1.7)	1.01 (0.22-4.55)	0.994
09:01	4 (1.7)	2 (1.1)	0.67 (0.12-3.68)	0.640
10:01	10 (4.2)	2 (1.1)	0.31 (0.07-1.47)	0.121
12:01	5 (2.1)	2 (1.1)	0.53 (0.10-2.76)	0.445
13:01	8 (3.4)	13 (7.4)	2.81 (1.12-7.00)	0.021[a]
13:02	10 (4.2)	8 (4.6)	1.08 (0.42-2.79)	0.880
14:01	3 (1.3)	2 (1.1)	0.89 (0.15-5.40)	0.902
14:02	17 (7.2)	2 (1.1)	0.15 (0.03-0.65)	0.004[a]
15:01	10 (4.2)	6 (3.4)	0.80 (0.28-2.23)	0.667
15:03	5 (2.1)	4 (2.3)	1.07 (0.28-4.06)	0.916
16:02	13 (5.5)	7 (4.0)	1.41 (0.55-3.60)	0.474

*HLA-DQA1**	PNR (N=131)	PR (N=81)	OR (95% CI)	p-value
01:01	34 (13.0)	16 (9.9)	0.73(0.39-1.38)	0.336
01:02	36 (13.8)	23 (14.3)	1.04 (0.59-1.83)	0.895
01:03	8 (3.1)	17 (10.6)	3.72 (1.57-8.83)	0.002[a]
02:01	26 (10.0)	20 (12.4)	1.28 (0.69-2.37)	0.436
03:01	48 (18.4)	33 (20.5)	1.14 (0.69-1.89)	0.602
04:01	33 (12.6)	19 (11.8)	0.92 (0.50-1.68)	0.791

05:01	18 (6.9)	6 (3.7)	0.52 (0.20-1.34)	0.170
05:03	18 (6.9)	3 (1.9)	0.26 (0.07-0.88)	0.021[a]
05:05	36 (13.8)	22 (13.7)	0.99 (0.56-1.75)	0.963
HLA-DQB1*	**PNR (N=131)**	**PR (N=81)**	**OR (95% CI)**	**p-value**
02:01	19 (7.3)	11 (6.8)	0.93 (0.43-2.01)	0.857
02:02	20 (7.6)	19 (11.7)	1.61 (0.83-3.11)	0.156
03:01	59 (22.5)	26 (16.0)	0.66 (0.40-1.10)	0.106
03:02	39 (14.9)	26 (16.0)	1.09 (0.64-1.88)	0.747
03:03	12 (4.6)	5 (3.1)	0.66 (0.23-1.92)	0.446
04:02	28 (10.7)	18 (11.1)	1.04 (0.56-1.96)	0.891
05:01	32 (12.2)	13 (8.0)	0.63 (0.32-1.23)	0.174
06:02	25 (9.5)	15 (9.3)	0.97 (0.49-1.89)	0.923
06:03	7 (2.7)	14 (8.6)	3.45 (1.36-8.73)	0.006[a]
06:04	7 (2.7)	4 (2.5)	0.92 (0.27-3.20)	0.899
06:09	1 (0.4)	3 (1.9)	4.92 (0.51-47.74)	0.128

After 12 months of follow-up, ELISA results allowed classify individuals as persistent non-responder (PNR) or persistent responder (PR), as described in legend of Fig.1. HLA class II alleles frequencies less than 1% were not included to this descriptive analysis; each individual (N=212) corresponded to two observations (N=424; two HLA class II alleles each). [a] Statistically significant differences ($p<0.05$, Qui-square test or Fisher's exact test as appropriate).
