# Supplementary material for: The Presence, Persistence and Functional Properties of Plasmodium vivax Duffy Binding Protein II Antibodies Are Influenced by HLA Class II Allelic Variants
Source: PLoS Negl Trop Dis. 2016 Dec 13;10(12):e0005177. doi: 10.1371/journal.pntd.0005177 (PMC5154503; doi:10.1371/journal.pntd.0005177)
Supplement: S3 Table — (PDF) [file pntd.0005177.s007.pdf]

S3 Table. Association between binding inhibitory antibody (BIAbs) response against *P. vivax* Duffy binding protein region II (DBPII) and HLA class II (*DRB1, DQB1* and *DQA1*) alleles of individuals naturally exposed to malaria

	DBPII BIAbs response			
HLA-DRB1*	**Negative (N=95) n (%)**	**Positive (N=48) n (%)**	**OR (95% CI)**	**p-value**
01:02	6 (3.2)	4 (4.2)	1.35 (0.37-4.90)	0.649
03:01	13 (6.8)	4 (4.2)	0.60 (0.19-1.89)	0.377
04:03	4 (2.1)	4 (4.2)	2.04 (0.50-8.40)	0.311
04:04	6 (3.2)	3 (3.1)	1.00 (0.24-4.10)	1.000
04:05	9 (4.7)	2 (2.1)	0.43 (0.09-2.05)	0.278
04:07	3 (1.6)	1 (1.0)	0.66 (0.07-6.49)	0.722
04:11	13 (6.8)	6 (6.3)	0.92 (0.34-2.50)	0.866
07:01	15 (7.9)	16 (16.7)	2.36 (1.10-5.06)	0.022[a]
08:01	2 (1.1)	2 (2.1)	2.02 (0.28-14.6)	0.477
08:02	10 (5.3)	5 (5.2)	1.00 (0.33-3.01)	1.000
08:04	14 (7.4)	2 (2.1)	0.27 (0.6-1.23)	0.069
08:07	2 (1.1)	3 (3.1)	3.06 (0.50-18.8)	0.203
09:01	4 (2.1)	1 (1.0)	0.49 (0.05-4.51)	0.524
10:01	6 (3.2)	1 (1.0)	0.32 (0.04-2.77)	0.280
11:01	10 (5.3)	7 (7.3)	1.43 (0.52-3.90)	0.480
11:04	3 (1.6)	1 (1.0)	0.66 (0.07-6.49)	0.722
12:01	4 (2.1)	3 (3.1)	1.51 (0.33-6.94)	0.589
13:01	8 (4.2)	6 (6.3)	1.53 (0.51-4.57)	0.439
13:02	12 (6.3)	6 (6.3)	1.00 (0.36-2.75)	1.000
14:02	10 (5.3)	3 (3.1)	0.59 (0.16-2.19)	0.422
15:01	9 (4.7)	3 (3.1)	0.66 (0.17-2.48)	0.532
15:03	1 (0.5)	2 (2.1)	4.06 (0.36-45.9)	0.219
16:02	9 (4.7)	2 (2.1)	0.43 (0.09-2.05)	0.278
HLA-DQA1*	**Negative (N=104)**	**Positive (N=58)**	**OR (95% CI)**	**p-value**
01:01	20 (9.6)	11 (9.5)	0.98 (0.45-2.13)	0.969
01:02	27 (13.0)	16 (13.8)	1.07 (0.55-2.08)	0.837
01:03	9 (4.3)	8 (6.9)	1.63 (0.61-4.38)	0.321
02:01	17 (8.2)	22 (19.0)	2.62 (1.31-5.24)	0.004[a]
03:01	47 (22.6)	22 (19.0)	0.80 (0.45-1.41)	0.445
04:01	29 (13.9)	14 (12.1)	0.85 (0.43-1.68)	0.634
05:01	14 (6.7)	5 (4.3)	0.62 (0.22-3.78)	0.375

05:03	12 (5.8)	5 (4.3)	0.73 (0.25-2.14)	0.573
05:05	31 (14.9)	12 (10.3)	0.66 (0.32-1.34)	0.247
HLA-DQB1*	**Negative (N=105)**	**Positive (N=58)**	**OR (95% CI)**	**p-value**
02:01	19 (9.0)	7 (6.0)	0.64 (0.26-1.58)	0.331
02:02	15 (7.1)	20 (17.2)	2.69 (1.30-5.55)	0.005[a]
03:01	45 (21.4)	18 (15.5)	0.67 (0.37-1.22)	0.189
03:02	42 (20.0)	17 (14.7)	0.68 (0.37-1.27)	0.223
03:03	6 (2.9)	6 (5.2)	1.84 (0.58-5.88)	0.292
04:02	25 (11.9)	14 (12.1)	1.01 (0.50-2.03)	0.977
05:01	18 (8.6)	11 (9.5)	1.11 (0.50-2.44)	0.792
06:02	18 (8.6)	8 (6.9)	0.79 (0.33-1.87)	0.586
06:03	8 (3.8)	7 (6.0)	1.61 (0.57-4.58)	0.364
06:04	8 (3.8)	4 (3.4)	0.90 (0.26-3.05)	0.862

Functional assays to detect DBPII BIAbs allow individual classification as positive (inhibitory binding > 50%) or negative (≤ 50%) for the presence of antibodies. Only alleles with frequency ≥ 1% were analyzed; each individual contributed with two HLA class II alleles. [a]Statistically significant differences (p<0.05; Qui-square test or Exact Fisher test as appropriate).
